# The Role of Post-Translational Modifications in Necroptosis

**DOI:** 10.3390/biom15040549

**Published:** 2025-04-09

**Authors:** Hao Xiao, Zeping Han, Min Xu, Xukang Gao, Shuangjian Qiu, Ning Ren, Yong Yi, Chenhao Zhou

**Affiliations:** 1 Department of Liver Surgery and Transplantation, Zhongshan Hospital, Fudan University, Shanghai 200032, China; 24111210116@m.fudan.edu.cn (H.X.); 24211210041@m.fudan.edu.cn (Z.H.);; 2 Liver Cancer Institute, Zhongshan Hospital, Fudan University, Shanghai 200032, China; 3 Key Laboratory of Carcinogenesis and Cancer Invasion, Fudan University, Ministry of Education, Shanghai 200032, China

**Keywords:** necroptosis, post-translational modifications (PTMs), targeted therapy, mechanism

## Abstract

Necroptosis, a distinct form of regulated necrosis implicated in various human pathologies, is orchestrated through sophisticated signaling pathways. During this process, cells undergoing necroptosis exhibit characteristic necrotic morphology and provoke substantial inflammatory responses. Post-translational modifications (PTMs)—chemical alterations occurring after protein synthesis that critically regulate protein functionality—constitute essential regulatory components within these complex signaling cascades. This intricate crosstalk between necroptotic pathways and PTM networks presents promising therapeutic opportunities. Our comprehensive review systematically analyzes the molecular mechanisms underlying necroptosis, with particular emphasis on the regulatory roles of PTMs in signal transduction. Through systematic evaluation of key modifications including ubiquitination, phosphorylation, glycosylation, methylation, acetylation, disulfide bond formation, caspase cleavage, nitrosylation, and SUMOylation, we examine potential therapeutic applications targeting necroptosis in disease pathogenesis. Furthermore, we synthesize current pharmacological strategies for manipulating PTM-regulated necroptosis, offering novel perspectives on clinical target development and therapeutic intervention.

## 1. Background

Cell death holds unique physiological and pathological implications. The maintenance of organismal homeostasis depends on the precise regulation of cell death, proliferation, and differentiation [1]. Based on their regulatory mechanisms, cell death can be classified into two major categories: regulated cell death (RCD) and accidental cell death (ACD) [2,3,4,5]. RCD encompasses multiple forms, including apoptosis, necroptosis, autophagy, ferroptosis, cuproptosis, pyroptosis, and NETosis, while ACD primarily refers to necrosis. Notably, necroptosis represents a unique form of RCD that shares morphological features with necrosis, characterized by plasma membrane rupture and the subsequent release of intracellular components [4].

Extensive research has established significant associations between necroptosis and various pathological conditions, including tumorigenesis [6], inflammation disorders [7], neurodegenerative diseases [8], cerebrovascular accidents [9], myocardial infarction [10], aortic aneurysm [11], and respiratory diseases [12]. This caspase-independent form of regulated cell death is governed by intricate signaling pathways, with RIPK1 (Receptor-interacting protein kinase 1), RIPK3 (Receptor-interacting protein kinase 3), and MLKL (Mixed lineage kinase domain-like protein) serving as core regulatory components. The initiation of necroptosis is triggered through specific receptors, notably death receptors (DRs) and pattern recognition receptors (PRRs), along with their associated signaling molecules. Under conditions of caspase-8 inhibition, RIPK1 undergoes autophosphorylation and subsequently recruits and activates RIPK3 through phosphorylation. This interaction leads to the formation of the ripoptosome complex. The assembly progresses with the recruitment and phosphorylation of MLKL, resulting in necrosome formation. Ultimately, MLKL oligomerization facilitates membrane translocation, culminating in cellular membrane disruption and cell death [13,14].

In such a sophisticated signaling cascade, post-translational modifications (PTMs) profoundly influence the progression of necroptosis. PTMs represent essential biochemical processes that modulate protein function through structural alterations, including proteolytic cleavage and side-chain modifications, ultimately affecting protein conformation and activity [15]. While phosphorylation and ubiquitination have been most extensively studied in necroptotic regulation, emerging evidence highlights the involvement of additional modifications. These include methylation, nitrosylation, glycosylation, acetylation, caspase-mediated cleavage, disulfide bond formation, and SUMOylation, collectively orchestrating the necroptotic process.

A comprehensive understanding of necroptosis and its associated post-translational modifications (PTMs) is essential for developing targeted therapeutic approaches. Deciphering the intricate interplay between necroptotic pathways and PTM networks may reveal novel therapeutic targets and intervention strategies, particularly for diseases that currently lack effective treatments. This review systematically examines the molecular mechanisms underlying necroptosis, followed by a detailed analysis of the specific PTMs involved in its regulation. Furthermore, we address current controversies and knowledge gaps in the field, including emerging molecular mechanisms, PTM-related therapeutic targets, and future research directions. Through this comprehensive analysis, we aim to provide both a current perspective and a roadmap for future investigations in this rapidly evolving field.

## 2. Current Knowledge of Necroptosis

Necroptosis represents a distinct form of programmed cell death characterized by unique mechanisms, pathological consequences, and clinical significance. Unlike immunologically silent apoptosis, necroptosis triggers robust inflammatory responses through plasma membrane rupture and the release of damage-associated molecular patterns (DAMPs) [2]. This inflammatory cascade exceeds even that of pyroptosis, which releases IL-1β and IL-18 through Gasdermin pores [16]. The impact of necroptosis extends beyond the single-cell precision of apoptosis, often causing spreading tissue damage as seen in ischemia–reperfusion injury. This contrasts with ferroptosis, which primarily affects lipid peroxidation-sensitive cells such as renal tubular epithelia [17]. Temporally, necroptosis is completed within 4–6 h following tumor necrosis factor α (TNFα) stimulation combined with cIAP inhibitors or Z-VAD-FMK, positioning it between rapid pyroptosis (under 30 min) and slower ferroptosis (24–48 h) [17,18,19,20].

Molecularly, necroptosis depends on RIPK1 autophosphorylation and necrosome formation with RIPK3, ultimately activating MLKL to form membrane pores [21]. This pathway differs fundamentally from the inflammasome–caspase–Gasdermin axis in pyroptosis and the system Xc^−^/GSH/GPX4 pathway in ferroptosis [16,22]. Morphologically, necroptotic cells exhibit swelling and organelle damage alongside partial chromatin condensation, without the cellular shrinkage or blebbing seen in apoptosis [19].

These regulated cell death mechanisms share upstream signals such as TNF receptor family activation while serving as important therapeutic targets. The critical role of necroptosis in stroke, myocardial infarction, inflammatory bowel disease, and viral infections has made it a focus of modern medical research [18].

The initiation of necroptosis is mediated by diverse upstream signals, such as death receptors (including TNFR1, FAS (also known as CD95 or APO-1), DR3 (also known as TRAMP or APO-3), TRAILR1 (also known as DR4), TRAILR2 (also known as DR5, TRICK, or KILLER), and DR6) [23]; pattern recognition receptors (including Toll-like receptors (TLR4 and TLR3)) [24]; reactive oxygen species (ROS); Z-DNA binding protein 1 (ZBP1); and cytosolic nucleic acid sensors such as RIG-I and STING, which together form the upstream signaling of the necroptosis pathway. These signals converge to activate a conserved molecular cascade: RIPK1 autophosphorylation, RHIM-mediated RIPK1–RIPK3 interaction, and subsequent RIPK3 oligomerization, which phosphorylates MLKL. Phosphorylation plays two pivotal roles in MLKL activation: (i) facilitating conformational transitions and (ii) mediating membrane translocation. Phosphorylated MLKL undergoes a structural shift from an autoinhibited state to an active conformation, forming dimers via its pseudokinase domain. This transition releases the brace region into an elongated helix, which subsequently oligomerizes into trimers or tetramers through coiled-coil assembly, exposing the 4-helical bundle domain (4HBD) [25,26]. Following tetramerization, MLKL dissociates from the necrosome and interacts with molecular chaperones (e.g., Hsp90 and Hsp70), facilitating its translocation to the plasma membrane. The exposed 4HB domain then binds to membrane phosphatidylinositol phosphates (PIPs), compromising membrane integrity and triggering DAMP release [27,28,29,30]. Additionally, MLKL pores may permit ion influx (e.g., Ca²⁺, Na⁺), disrupting osmotic balance and contributing to cellular swelling and rupture [28,31].

Beyond plasma membrane targeting, MLKL interacts with mitochondrial proteins to amplify necroptotic signaling. At mitochondria, MLKL binds to phosphoglycerate mutase family member 5 (PGAM5), which stabilizes MLKL through dephosphorylation, enhancing its oligomerization and membrane-disrupting activity [32,33,34]. Concurrently, MLKL can also interact with dynamin-related protein 1 (DRP1), a downstream molecule of PGAM5, inducing mitochondrial fission that releases more ROS to exacerbate necroptosis [34]. Additionally, mitochondrial DNA (mtDNA) can act as a DAMP to further amplify inflammatory responses by activating the cGAS-STING pathway [35,36]. Additionally, ATP depletion resulting from mitochondrial dysfunction impairs membrane repair mechanisms, enhancing pore formation efficiency [37]. These coordinated interactions significantly accelerate necroptosis progression.

The following sections will systematically examine these upstream signals and their downstream effectors, providing a comprehensive understanding of necroptosis induction mechanisms, including the molecular details of MLKL activation, oligomerization, and membrane targeting.

### 2.1. TNFR1

**Figure 1:**
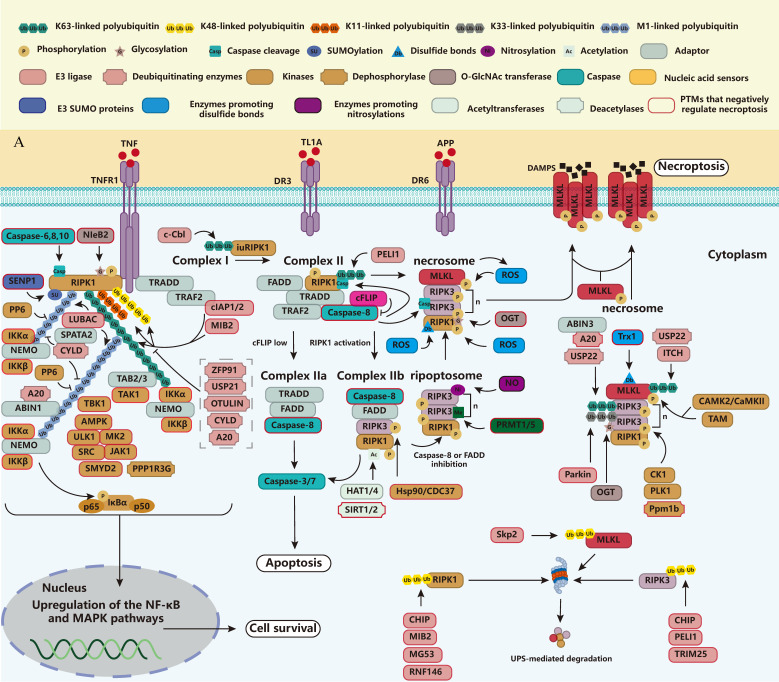

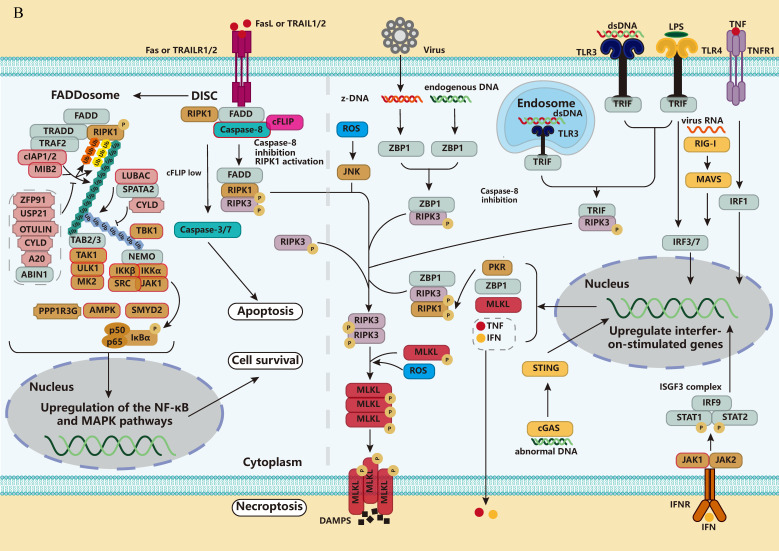
The signaling pathway of necroptosis and its PTMs. (**A**) Binding of cell surface TNFR1, DR3, DR6, and their respective ligands TNF, TLA1, and APP recruits the formation of Complex I, which promotes the activation of the NF-κB and MAPK pathways, the expression of inflammatory genes, and cell survival. After Complex I is destabilized, it can form Complex II, including Complex IIa and IIb, which induce apoptosis and RIPK1-dependent apoptosis, respectively. When caspase-8 activity is inhibited, a ripoptosome can be formed in the cell, which further forms a necrosome and induces necroptosis. The PTMs of RIPK1, RIPK3, and MLKL and their corresponding modified proteins are also labeled in the figure. (**B**) On the left side of the figure, FAS and TRAILR1/2 bind to their respective ligands FASL and TRAIL1/2 to first form the death-inducing signaling complex (DISC), which can induce apoptosis and necroptosis when caspase-8 is inhibited. After separation from the receptor, a complex similar to Complex I can be formed in the cytoplasm as the FADDosome, which can promote the expression of the NF-κB and MAPK pathways and cell survival. On the right side of the figure, only caspase-8 inhibition is shown. When caspase-8 is not inhibited, TLR3, TLR4, and ZBP1 can also form Complex I-like complexes in the cytoplasm upon binding to their corresponding ligands, promoting cell survival. When caspase-8 is inhibited, TLR3 and TLR4 promote its autophosphorylation and the recruitment of MLKL through direct binding of TRIF to RIPK3, while ZBP1 binds to RIPK3 directly, which promotes necroptosis. Interferon (IFN) can promote necroptosis through multiple pathways.

TNF-TNFR1 signaling represents the most extensively characterized pathway in necroptosis research. As a member of the TNF receptor superfamily (TNFRSF), TNFR1 contains a cytoplasmic death domain (DD) that orchestrates inflammatory responses, apoptosis, and necroptosis [38,39,40]. Ligand binding initiates the recruitment of TNFRSF1A associated via death domain (TRADD) and RIPK1 through the DD, followed by the assembly of TNF receptor associated factor 2 (TRAF2) and the E3 ubiquitin ligases cellular inhibitor of apoptosis 1 and 2 (cIAP1/2). These components facilitate the attachment of K11-, K48-, and K63-linked polyubiquitin chains to RIPK1 and other signaling proteins, while also promoting the engagement of the linear ubiquitin chain assembly complex (LUBAC, consisting of heme-oxidized IRP2 ubiquitin ligase-1, HOIL-1L; HOIL-1-interacting protein, HOIP; and SHANK-associated RH domain-interacting protein, SHARPIN) [41,42]. TNFR1, TRADD, and RIPK1 are conjugated to the M1-linked polyubiquitin chain mediated via LUBAC, which facilitates the formation of the IkB kinase (IKK) complex consisting of IKKα, IKKβ, and NF-κB essential modulator (NEMO) and the TGF activating kinase 1 (TAK1)-TAK1 Binding Protein 2/3 (TAB2/3) Complex [43,44,45,46,47]. Activation of TAK1 and IKK induces the activation of NF-κB and mitogen-activated protein kinase (MAPK) [48]. These are the major components of the so-called Complex I (Figure 1A). Complex I favors pro-inflammatory gene expression and inhibits cell death. Other kinases in Complex I, such as TANK binding kinase 1 (TBK1), MAPK activated protein kinase 2 (MAPKAPK2, MK2), the tyrosine kinases JAK1 and SRC, ULK1, and adenylate-activated protein kinase (AMPK), can negatively regulate protein activity by phosphorylating RIPK1 [49,50,51,52,53,54,55].

The progression from Complex I to Complex IIa/b depends on multiple critical conditions. These include enhanced RIPK1 autophosphorylation or kinase activity, suppressed RIPK1 ubiquitination, inhibited RIPK1 phosphorylation, altered post-translational modifications (glycosylation, caspase cleavage, SUMOylation), and Complex I destabilization. Various molecular interventions disrupt this transition, such as IAP inhibitors, LUBAC inhibitors, RIPK1 autophosphorylation, RIPK1-DD-mediated dimerization, phosphorylase inhibitors, and kinase knockout [56]. Conversely, cylindromatosis (CYLD) facilitates this progression between complexes [57]. CYLD is linked to LUBAC via the adaptor protein spermatogenesis associated 2 (SPATA2), and it is able to eliminate M1- or K63-linked polyubiquitin, a process that can be detected by A20 and A20 binding and inhibitor of NF-kB (ABIN-1) [58,59,60,61]. Similarly, other deubiquitinating enzymes, such as USP21, OTU deubiquitinase with linear linkage specificity (OTULIN), and zinc finger protein 91 (ZFP91), can likewise also facilitate necroptosis [62,63,64,65,66,67,68]. TRAF2 can function as an inhibitory factor to suppress necroptosis signaling by (1) promoting Complex I stabilization and NF-κB activation by ubiquitinating RIPK1 through binding to cIAP1/2 and (2) inhibiting necrosome formation by binding directly to MLKL, a process that is inhibited by CYLD, providing a direct basis for the idea that CYLD can facilitate necroptosis [57]. Following the destabilization of Complex I, TRADD and RIPK1 dissociate from TNFR1 and translocate to the cytoplasm [69]. Both proteins, through their DDs, interact with FAS associated via death domain (FADD,) an adaptor protein that recruits caspase-8 [58]. This assembly, termed Complex IIa, primarily drives apoptotic signaling and consists of TRADD, FADD, and caspase-8 (Figure 1A). Complex IIa transforms into Complex IIb under specific molecular conditions: RIPK1 ubiquitination inhibition, enhanced autophosphorylation, or stimulated kinase activity. Complex IIb comprises RIPK1, RIPK3, FADD, and caspase-8, initiating RIPK1 kinase activity-dependent apoptosis (RDA) (Figure 1A). The inhibition of caspase-8 activity promotes necrosome formation, resulting in necroptosis rather than apoptosis. Caspase-8 functions as a critical regulatory switch between these cell death pathways. Any agent preventing the caspase-8-mediated cleavage of RIPK1 and RIPK3 facilitates the apoptosis-to-necroptosis transition. At low concentrations, the heterodimerization of cFLIPL (cellular FLICE-inhibitory protein, long isoform) with caspase-8 activates caspase-8 through cleavage, thereby preventing necrosome formation and halting necroptosis. Conversely, normal or elevated cFLIPL levels inhibit both caspase-8 activity and subsequent apoptotic and RDA processes [70,71,72].

### 2.2. Fas or TRAILR1/2

Upon the binding of FASL and TRAILR1/2 to their corresponding ligands, FADD and caspase-8 are subsequently recruited to form the death-inducing signaling complex (DISC), which cleaves caspase-3/7 and triggers apoptosis [73,74]. Similar to TNFR1 signaling in Complex IIb, DISC can likewise bind to RIPK1, leading to the onset of RDA. When caspase-8 activity is inhibited, cell necroptosis can be triggered further [75]. In the cytoplasm, FADD and caspase-8 assemble a multiprotein complex resembling Complex I, termed the “FADDosome.” This structure incorporates TRADD, TRAF2, cIAP1/2, RIPK1, TAK1, and the IKK complex, thereby activating the NF-κB and MAPK pathways to initiate inflammatory responses [76]. Not surprisingly, in contrast to TNFR1-induced signals, the FAS- and TRAILR1/2-induced signals form DISCs to trigger the initiation of death before the formation of intracytoplasmic complexes to trigger inflammatory signaling (Figure 1B).

### 2.3. DR3 and DR6

Death receptor 3 (DR3, also known as TNFRSF25) was originally identified as an essential T cell co-stimulatory molecule [77]. In addition to apoptosis, DR3 can mediate necrosome formation in the presence of its ligand TL1A [78] (Figure 1A). In allergic inflammation, such as asthma, TL1A-DR3-induced necroptosis may be one of the major causes of exacerbation [79]. TL1A inhibitors have demonstrated therapeutic efficacy in inflammatory bowel disease patients, highlighting the potential significance of necroptosis in disease pathogenesis [80].

Death Receptor 6 (DR6) is extensively represented in a diverse range of cells, including neuronal cells and endothelial cells [81]. The binding of amyloid precursor protein (APP) to DR6 elicits necroptosis [82] (Figure 1A). Tumor cells can induce endothelial cell necroptosis by secreting APP, which in turn facilitates their metastasis [83]. Thus, it is apparent that all DRs are inextricably intertwined with necrotic apoptosis.

### 2.4. TLR3/4 and ZBP1

TLR3 is stimulated by polyinosine–polycytidylic acid (poly(I:C)), dsRNA, and vRNA, whereas TLR4 is upregulated by lipopolysaccharide (LPS), CD14, or myeloid differentiation primary response 88 (MyD88) [84,85]. TLR3 and TLR4 initiate necroptotic signaling by engaging RIPK1 and RIPK3 through Toll/IL-1 receptor domain-containing adaptor inducing interferon-beta (TRIF), which interacts with the RHIM domain. This assembly further recruits TRADD, TRAF2, cIAP1/2, LUBAC, and the FADD-caspase-8 complex, ultimately activating the NF-κB and MAPK pathways [86]. When ubiquitination and phosphorylation are inhibited, this complex can trigger apoptosis, and then caspase-8 activity, when further inhibited, can initiate necrosome formation and necroptosis [87] (Figure 1B).

ZBP1 (also known as DNA-dependent activator of IFN regulatory factors (DAI)) possesses a RHIM structural domain that binds to RIPK3, inducing the phosphorylation of RIPK3 and subsequent phosphorylation of MLKL, leading to necroptosis, independently of RIPK1 [88] (Figure 1B). ZBP1 further triggers cellular inflammation via RIPK1–FADD–caspase-8-induced apoptosis [89]. Notably, ZBP1 mediates nucleus-to-cytoplasm necroptosis. Influenza A virus (IAV) replication produces z-RNAs that activate ZBP1 within the nucleus of infected cells. This activation triggers RIPK3-dependent MLKL phosphorylation, resulting in nuclear membrane disruption, cytosolic DNA release, and subsequent necroptosis [90]. However, it has been demonstrated that the RHIM of RIPK1 inhibits ZBP1 from binding and the activation of RIPK3, further inhibiting ZBP1-induced necroptosis [91]. Recent investigations have demonstrated that RIPK1-DD inhibits ZBP1- and TRIF-induced necroptosis, which is mediated by the cleavage of RIPK1 and RIPK3 by FADD-caspase-8, which is recruited by RIPK1-DD, inhibiting its activation [92].

### 2.5. ROS

ROS are ubiquitous in the development of necroptosis and are primarily generated by the NOX family and mitochondria. ROS have been implicated in facilitating RIPK1 autophosphorylation, thereby promoting RIPK1 and RIPK3 to generate the necrosome for necroptosis [93,94,95,96,97]. The potential mechanism involves ROS facilitating the oxidation of the RIPK1 C257, C268, and C586 cysteines to generate disulfide bonds to facilitate RIPK1 polymerization, which would promote RIPK1 S161 autophosphorylation and enhanced affinity for RIPK3 [94]. ROS can also facilitate RIPK3-MLKL production [98] or p-MLKL oligomerization independently [99]. ROS can also activate RIPK3-dependent necroptosis production through the c-Jun N-terminal kinase (JNK) pathway [100,101]. Conversely, ROS production is modulated by the RIPK1-RIPK3 complex, which facilitates ROS production [102]. RIPK3 can also facilitate ROS production through the JNK pathway [103]. TRADD has been demonstrated to bind specifically to RIPK3 to trigger ROS production and necroptosis, a process that is independent of RIPK1 [104]. Emerging evidence suggests that mitochondrial ROS (mtROS) play a pivotal role in the transition from pyroptosis to necroptosis. In macrophages carrying the leucine-rich repeat kinase 2 (*LRRK2^G2019S^*) mutation, bacterial infection triggers inflammasome activation, resulting in the caspase-1-mediated cleavage of GSDMD and pro-IL-1β. Unlike typical pyroptosis, GSDMD pores localize to mitochondria rather than the plasma membrane, causing mitochondrial damage. This aberrant localization promotes mtDAMP release, mtROS production, and subsequent RIPK1/RIPK3 activation, thereby shifting the cell death modality toward necroptosis [105] (Figure 1A,B).

### 2.6. IFN

Type I and II interferons (IFNs) promote necrosome assembly under two distinct conditions: (1) inhibition or loss of FADD (via S194 phosphorylation) or caspase-8 activity, and (2) activation of the viral RNA-sensing kinase (PKR, protein kinase R), which interacts with RIPK1 to facilitate necrosome formation [106]. Additionally, cytoplasmic nucleic acid sensors such as RIG-I and STING induce IFN-I and TNFα production, creating an autocrine loop that amplifies necroptotic signaling [107,108]. IFN-I activates the ISGF3 complex (STAT1-STAT2-IRF9) through IFNAR1 binding, leading to transcription-dependent necrosome activation [109]. Similarly, the TNF-IRF1 and LPS-IRF3/7 signaling pathways stimulate IFNβ production, further reinforcing this autocrine feedback mechanism (Figure 1B) [109].

Overall, the conditions under which necroptosis occurs are more demanding, and caspase inactivation is generally observed after viral infection, so we can envisage that in the natural situation, necroptosis is a complementary death pathway in response to viral infection, in which caspase-8 mediates the conversion of apoptosis to necroptosis.

## 3. Post-Translational Modifications in Necroptosis

### 3.1. Ubiquitination

Ubiquitination forms a central regulatory framework underlying necroptotic pathways. This post-translational modification involves the covalent attachment of ubiquitin (Ub) molecules to substrate proteins, typically linking the C-terminal glycine residue (Gly76) of ubiquitin to lysine side chains on target proteins. The ubiquitination cascade proceeds through a three-enzyme relay: E1 (ubiquitin-activating enzyme) initiates the process through ATP-dependent ubiquitin activation, E2 (ubiquitin-conjugating enzyme) carries the activated ubiquitin, and E3 (ubiquitin ligase) facilitates the final transfer to specific substrate proteins. This sophisticated enzymatic machinery enables the precise control over necroptotic signaling components, influencing their stability, activity, and interaction dynamics [110]. The removal of ubiquitin molecules is mediated by the DeUBiquitinating enzyme (DUB) family. The linkage of ubiquitin can be classified as monoubiquitination and polyubiquitination. Monoubiquitination can be subdivided into monoubiquitination and multi-monoubiquitination, which refers to the attachment of a single Ub molecule to a target protein or the attachment of multiple Ub molecules to multiple lysines (K) of a target protein. Polyubiquitination means that Ub can polymerize to form a chain or branching structures. Ub contains seven lysines (K6, K11, K27, K29, K33, K48, K63) and an N-terminal methionine (M1), and when a specific residue links all the Ub monomers together, the chain is named after that residue, such as M1-linked chains. Chains composed of different Ub residues produce different spatial conformations, which in turn affect their functions. The chains connected by M1- and K63- adopt a linear chain-like structure, whereas the chains connected by K48- have a zig-zag spherical structure. Different ubiquitin linkage types orchestrate distinct cellular fates. K63- and M1-linked chains primarily facilitate protein complex assembly and signaling platform formation. In contrast, K48-linked chains serve as canonical degradation signals, marking proteins for recognition and proteolysis by the ubiquitin proteasome system (UPS) [111]. It is noteworthy that Ub molecules allow the formation of mixed chains, which refers to the fact that, for example, they can form K63-linked chains before being modified by M1-linked chains, thereby altering their biological function [112].

#### 3.1.1. RIPK1

**Figure 2:**
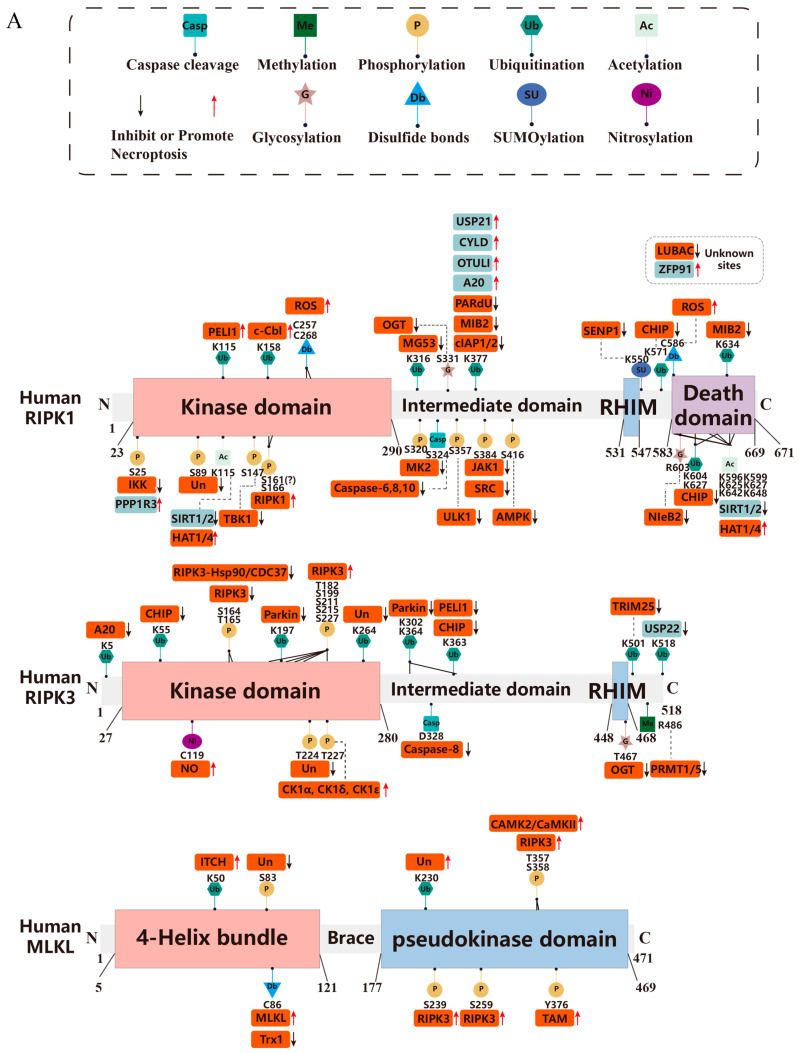

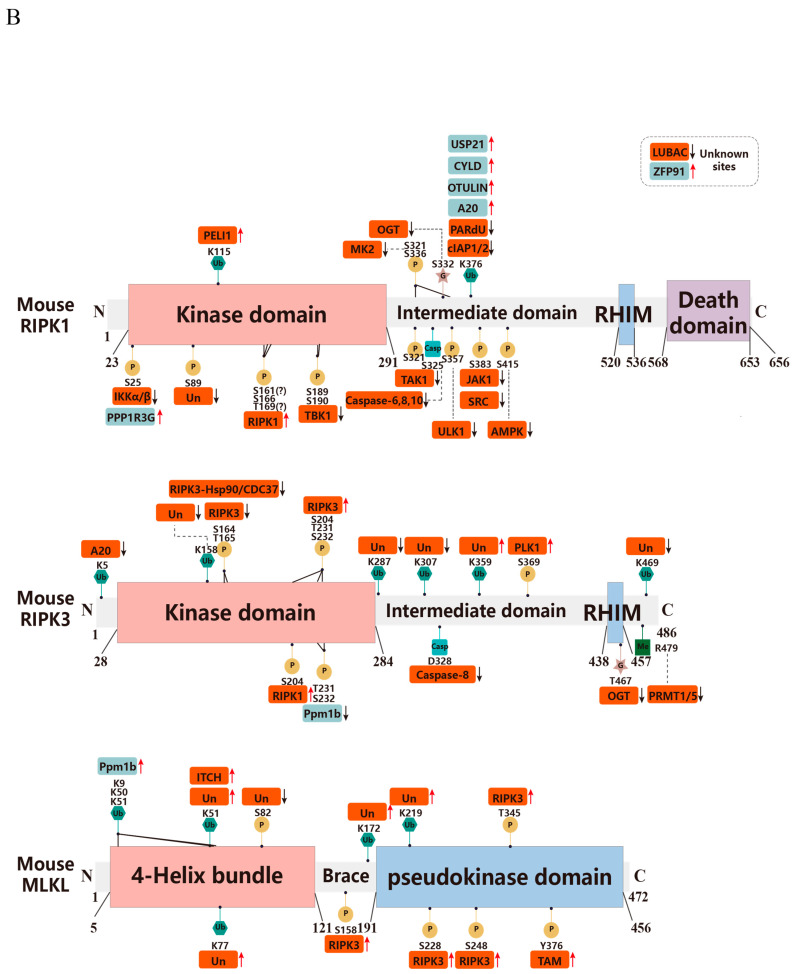
The structure of RIPK1, RIPK3, and MLKL of humans and mice and their respective PTM sites and their contribution to necroptosis. (**A**) PTMs of human RIPK1, RIPK3 and MLKL. (**B**) PTMs of mouse RIPK1, RIPK3 and MLKL. PTMs at different structural domains with different sites are labeled in the figure. Red boxes indicate proteins with added PTMs, blue boxes indicate proteins with removed PTMs, and arrow color and direction indicate contribution to necroptosis.

The modification of RIPK1 by ubiquitination regulates cell fate, determining whether it survives, whether pro-inflammatory signaling pathways are activated, or whether it dies. RIPK1 comprises multiple structural domains. These include the middle RHIM domain, the C-terminal DD, and the N-terminal serine/threonine kinase domain [69] (Figure 2A,B). The RHIM structural domain is responsible for its interaction with other proteins that possess RHIM, while the serine/threonine kinase domain possesses kinase activity that phosphorylates other proteins, and through the DD, stimulated by various signals, it can interact with other proteins possessing a DD such as TRADD, FADD, and caspase-8, to form Complex I.

The E3 ligases cIAP1 and cIAP2 serve as pivotal molecular regulators by catalyzing the K11-, K48-, and K63-linked polyubiquitination of RIPK1 and facilitating its recruitment to Complex I via TRADD-TRAF2 interactions. Specifically, these molecules modify RIPK1 at residue K377 through K63-linked ubiquitination, enabling subsequent LUBAC binding and NF-κB pathway activation [42]. This K63-linked modification is crucial for Complex I stabilization. SMAC mimetics, which inhibit cIAP1 and cIAP2, predictably promote the transition from Complex I to Complex IIa/b. Beyond K63-linked modifications, K48-linked polyubiquitination also regulates RIPK1 function. This regulation depends on cIAP1’s ubiquitin-associated (UBA) domain, which simultaneously suppresses RIPK1 kinase activity and promotes its K48-linked polyubiquitination-mediated degradation. These mechanisms effectively prevent the progression from Complex I to Complex II and block subsequent death signaling pathways [113]. In mice, K376 of RIPK1 (corresponding to K377 in humans) seems to be the primary site targeted by cIAP1/2 for ubiquitination. When RIPK1 has a K376R mutation, it changes the formation of the TNFR1 complex. This mutation also decreases the K11, K63, and linear ubiquitination of RIPK1, ultimately resulting in the death of mouse embryos [114]. LUBAC, another E3 ligase, consists of HOIL-1L, HOIP, and SHARPIN and is linked to Complex I via a K63-linked polyubiquitination chain added by cIAP1/2 [44,45,46,47]. LUBAC interacts with RIPK1 and NEMO via a linear polyubiquitination chain and can provide M1-linked chains for TRADD, TNFR1, and RIPK1, which provide attachment sites for the IKK complex, TAK1 complex, and TBK1, among others, which can further phosphorylate RIPK1 and stabilize Complex I [115,116]. Notably, PP6 is a member of the PPP family of serine/threonine protein phosphatases and has been suggested as a possible member of the novel Complex I that promotes necroptosis by negatively regulating LUBAC-mediated M1-linked polyubiquitination, facilitating RIPK1 activation and cFLIP degradation [117]. There is also an E3 ubiquitin ligase, Mind Bomb-2 (MIB2), which modifies the RIPK1 C-terminal portions K377 and K634 by adding K11-, K48-, and K63-linked polyubiquitination, disrupting RIPK1 oligomerization and the RIPK1-FADD association and inhibiting death signaling [118]. Poly ADP-ribosylation (PARylation) and PARylation-dependent ubiquitination (PARdU)-mediated ubiquitination occur on mouse RIPK1 K376 in response to necroptosis signaling stimulation. PARdU of RIPK1 is mediated by poly(ADP-ribose) polymerase 5A (PARP5A) and the E3 ubiquitin ligase RING finger protein 146 (RNF146), which interact multivalently to form a fluid cohesive complex that is recruited to activated RIPK1 by Tax1-binding protein 1 (TAX1BP1) to promote the proteasomal degradation of RIPK1 and inhibit necroptosis [119]. Mitsugumin 53 (MG53), an E3 ubiquitin ligase, attaches multiple ubiquitin chains to RIPK1 at residues K316, K604, and K627. This leads to the proteasome-mediated degradation of RIPK1, thereby inhibiting necroptosis [120].

Conversely, DeUBiquitinating enzymes can remove ubiquitination modifications and thereby regulate cell survival or death, including A20 (and its binding protein ABIN1), CYLD, USP21, and OTULIN. They can remove K63- or M1-linked polyubiquitination on human RIPK1 K377(mouse K376), leading to Complex I destabilization that triggers death signaling [62,63,64,65,66,67]. Zinc finger protein 91 (ZFP91) also induces RIPK1 deubiquitination to stabilize RIPK1 to promote necroptosis, but its specific site is unknown [68].

Ubiquitination can not only inhibit death signaling, but it can also promote death signaling. The E3 ubiquitin ligase Pellino 1 (PELI1) modifies K63-linked polyubiquitination chains on K115, which mediates RIPK1 activity and promotes the binding of activated RIPK1 to RIPK3 and MLKL to form a necrosome, leading to necroptosis [121]. However, the K115 mutation (present in both humans and mice) has no impact on RIPK1 ubiquitination. It also does not affect the TNF-stimulated NF-κB and MAPK signaling pathways [114]. Another E3 ligase, c-Cbl, modifies RIPK1 at K158 using K63-linked chains. When TAK1 is inhibited, LRRK2, anaphase-promoting complex subunit 11 (APC11), and c-Cbl are more strongly recruited to Complex I. This leads to the ubiquitination of RIPK1, forming a large, insoluble RIPK1 complex (iuRIPK1). This complex is an intermediate between Complex I and IIa/b and can trigger apoptosis or necroptosis [122]. The ubiquitination of RIPK1 in the cytoplasm but not in Complex I can also determine cell fate.

Under normal physiological circumstances, the E3 ligase carboxyl terminus of Hsp70-interacting protein (CHIP) binds to RIPK1 in the cytoplasm. This interaction leads to the ubiquitination of RIPK1 at K571, K604, and K627. Subsequently, the ubiquitin-proteasome system degrades RIPK1, thereby regulating its level of stability [123].

Overall, RIPK1 is degraded intracellularly in four ways at the same sites as mouse RIPK1 K376 or human RIPK1 K377: (1) UBA structural domain of cIAP1-mediated polyubiquitination [113]; (2) PARdU-mediated polyubiquitination [119]; (3) MG53-mediated polyubiquitination [120]; and (4) CHIP-mediated polyubiquitination [123]. Furthermore, in addition to PELI1- and c-Cbl-mediated ubiquitination, the ubiquitination of RIPK1 generally inhibits necroptosis by affecting its kinase activity and causing its proteasomal degradation (Figure 1A and Table 1).

#### 3.1.2. RIPK3

RIPK3 contains the RHIM structural domain and can interact with other proteins possessing the RHIM structural domain such as RIPK1, ZBP1, and TRIF to form a complex [14] (Figure 2A,B). The ubiquitination of RIPK3 at K5 stabilizes the RIPK1-RIPK3 complex. In contrast, the deubiquitinating enzyme A20 removes K63-linked polyubiquitination chains from RIPK3, which in turn inhibits necroptosis [139]. A20 binding and inhibitor of NF-kB 3 (ABIN3) can recruit A20 to enhance its inhibitory effect [140]. Mouse RIPK3-K469 ubiquitination restricts necroptosis and apoptosis by preventing upstream ubiquitination of K359, suggesting that the ubiquitination of K359 promotes necroptosis, but the enzymes that ubiquitinate it are not known [141]. The RIPK3 residues K158, K287, and K307 limit cell death in a way comparable to K469 [141].

Unlike A20, ubiquitin-specific peptidase 22 (USP22) is a DUB that deubiquitinates RIPK3 on K42, K351, and K518, but USP22 specifically promotes necroptosis through the deubiquitination of RIPK3 K518 [142].

In contrast, the following E3 ligases inhibit the proteasomal degradation of RIPK3 by adding K48-linked polyubiquitination that mediates its function. In the cytoplasm, RIPK3 K55 and K363 can be modified by the addition of ubiquitination by the aforementioned E3 ligase CHIP, leading to their lysosomal degradation and the negative regulation of necroptosis [123]. PELI1 acts as an E3 ligase that can add a K48-linked polyubiquitination to RIPK3 K363, whereas the phosphorylation of RIPK3 T182 leads to its interaction with PELI1, which, above all, results in the degradation of the kinase-active RIPK3 [143]. The E3 ligase TRIM25 directly interacts with RIPK3 via its SPRY domain. This interaction enables TRIM25 to mediate the addition of K48-linked polyubiquitination chains to RIPK3 at K501, which in turn promotes RIPK3 degradation [144]. RIPK3 K264 can be modified by K48-linked polyubiquitination and degraded by the UPS, but its E3 ligase is unknown [145].

Parkin is an E3 ligase. RIPK1/3 activates AMPK, which then phosphorylates Parkin at S9. This phosphorylation causes the polyubiquitination of RIPK3. Specifically, K33-linked polyubiquitination occurs at RIPK3 residues K197, K302, and K364. As a result, RIPK3 changes its conformation, inhibiting necrosome formation [146] (Figure 1A and Table 2).

#### 3.1.3. MLKL

MLKL consists of an N-terminal 4HB domain, a two-helix “brace” region, and a C-terminal pseudokinase domain. These three structural domains are crucial for MLKL’s killing effect [165] (Figure 2A,B). The ubiquitination of endogenous MLKL occurs on K51, K77, K172, and K219. RIPK3 phosphorylation of MLKL followed by a K63-linked polyubiquitination on K219 (human K230) prior to its translocation to the cell membrane can promote its oligomerization, ultimately leading to necroptosis, in which the sequence of phosphorylation and ubiquitination of MLKL is critical [166]. It has also been demonstrated that the ubiquitination of MLKL inhibits necroptosis. In the 4HB structural domain of MLKL, residues K9, K51, K69, and K77 are subject to ubiquitination modifications. Monoubiquitination of MLKL does not lead to cell death but instead promotes its proteasome- and lysosome-mediated degradation. In contrast, USP21, a DUB that removes all ubiquitination modifications on all of MLKL, enhances MLKL activation when it produces MLKL-USP21 fusion proteins with MLKL, and it can be assumed that the monoubiquitination of MLKL inhibits necroptosis [167]. Ubiquitination of MLKL aids in clearing intracellular bacteria. In human MLKL, K50, and in mouse MLKL, K50/51 can be modified by E3 ligase ITCH-mediated K63-linked polyubiquitination. This modification enables MLKL to bind to endosomal membranes. Subsequently, MLKL is exocytosed from the cell via extracellular vesicles. As a result, it enhances the disruption of intracellular bacteria by promoting the translocation of bacteria from the endosome to the lysosome [168]. Moreover, the E3 ligase S-phase kinase-associated protein 2 (Skp2) participates in the K48-linked polyubiquitination of MLKL, leading to its degradation. Yet, the precise modification site is still unclear [169].

Interestingly, the LUBAC-mediated M1-linked polyubiquitin modification does not act directly on MLKL, but rather, sways cell death by modulating the subcellular distribution of active MLKL [170] (Figure 1A and Table 3).

#### 3.1.4. FADD

FADD consists of a DD and death effector domain (DED), which plays a key role in constituting the DISC and interacting with caspase-8 [71]. The E3 ligase CHIP mediates the K6-linked polyubiquitination of FADD at K149 and K153. This process is crucial for preventing the formation of the DISC and thus inhibiting cell death [181]. In contrast, a distinct E3 ligase, Makorin ring finger protein 1 (MKRN1), promotes the proteasomal degradation of FADD. This action inhibits the formation of the DISC and death signaling. Nevertheless, the specific site of its action requires further study [182] (Table 4).

#### 3.1.5. Caspase-8

Procaspase-8 has two isoforms, procaspase-8a and procaspase-8b. These are activated mainly when two of their N-terminal death effector domains (DED 1 and DED 2) are recruited to the DISC and dimerized at the DED filaments formed there [214]. The two DEDs of procaspase-8a/b contain the large catalytic domain p18 and the small catalytic domain p10, respectively [214]. The E3 ligase Cullin 3 (CUL3) mediates the K48- and K63-linked polyubiquitination of K461 on the p10 subunit. This stabilizes the active caspase-8 heterotetramer. The process involves p62 binding to CUL3-ubiquitinated caspase-8 and forming an aggregated structure, enhancing procaspase-8 activity [192]. During endoplasmic reticulum (ER) stress, the E3 ligase TRIM13 promotes caspase-8 activity through K63-linked ubiquitination, but the specific ubiquitination site on caspase-8 is unknown [193].

Conversely, homologous to the E6-AP Carboxyl Terminus 3 (HECTD3) causes K63-linked polyubiquitination at caspase-8 K215. This does not lead to caspase-8 degradation but reduces its activation, promoting cell survival [194]. At the DISC, TRAF2 interacts with caspase-8, resulting in polyubiquitination at K224, K229, and K231 of the p18 structural domain. This leads to the proteasomal degradation of active caspase-8 and inhibits death signaling [195]. Similarly, the DR5-Cbl-b/c-Cbl-TRAF2 complex interacts with TRAF2. TRAF2 then mediates the K48-linked polyubiquitination of caspase-8, promoting its proteasomal degradation [215] (Table 4).

#### 3.1.6. cFLIP

The cFLIP proteins contain one long isoform called cFLIP_L_ and two short isoforms called cFLIP_S_ and cFLIP_R_ [216]. The cFLIP protein has two DED structural domains at the N-terminus, whereas cFLIP_L_ also has a catalytically inactivating cysteine asparaginase-like structural domain in its C-terminal region (p20 and p12) [216]. All types of cFLIP are widely recognized as antiapoptotic proteins, and when expressed at high levels, they compete with procaspase-8 for recruitment to the DISC via their DED domains. When expressed at low levels, cFLIPL exerts its pro-apoptotic function via the formation of a procaspase-8/cFLIPL heterodimer. In this heterodimer, cFLIPL stabilizes the active center of procaspase-8, thus enhancing caspase-8 activity. In contrast, cFLIPS and cFLIPR inhibit caspase-8 activation. They do so by forming an inactive heterodimer with procaspase-8, which prevents caspase-8 from being activated [217]. The HOIP catalytic subunit of the E3 ligase LUBAC forms M1-linked polyubiquitinated chains at K351 and K353 of cFLIP, which can inhibit K48-linked polyubiquitination, stabilize cFLIP, inhibit its degradation, and suppress the death process [205]. MIB2 may add K48- and K36-linked polyubiquitination at nine sites, namely, K351, K353, K381, K386, K389, K460, K462, K473, and K474, in cFLIP_L_, stabilizing cFLIP and inhibiting RIPK1 kinase activity and Complex II production [206]. Both the Skp1-Cullin-1-F-box (SCF) Cullin-Ring E3 ubiquitin ligase complex (SCF^Skp2^) with Skp2 and the E3 ubiquitin ligase ITCH can promote the degradation of cFLIP through ubiquitination. However, the precise ubiquitination site remains unclear [209,210]. The deubiquitinating enzyme (DUB) Usp27x inhibits cell death related to cFLIP. It does this by removing the K48-linked polyubiquitination added to cFLIP by the E3 ligase TRIM28. As a result, it blocks the proteasomal degradation of cFLIP [211] (Table 4).

#### 3.1.7. TNFR1

Upon activation, TNFR1 on the cell surface quickly recruits the E3 ligase RNF8. RNF8, along with the E2 ubiquitin-conjugating enzyme Ubc13, binds to TNFR1. RNF8 then modifies TNFR1 by adding K63-linked polyubiquitination to the receptor. This modification triggers the internalization of activated TNFR1, an essential step for cell death. Nevertheless, the precise site of this modification remains unconfirmed [186] (Table 4).

### 3.2. Phosphorylation

Protein phosphorylation, the addition of phosphate groups to proteins, is one of the most common and crucial post-translational modifications (PTMs). It is invariably involved in the necroptosis process [218]. Nine amino acid residues can be phosphorylated: serine, threonine, tyrosine, histidine, lysine, arginine, aspartic acid, glutamic acid, and cysteine. Depending on the amino acid residue, four types of phosphorylation can occur. Serine, threonine, or tyrosine hydroxyl groups undergo O-phosphorylation (forming a P-O bond). The nitrogen-containing side chains of histidine, lysine, or arginine experience N-phosphorylation (P-N bond). Aspartic acid or glutamic acid carboxyl groups are subject to A-phosphorylation (P-OCO bond), and cysteine sulphonyl groups participate in S-phosphorylation (P-S bond). Like ubiquitination, protein phosphorylation is regulated by phosphorylases and dephosphorylases. This regulation impacts cellular activities such as growth, development, death, and senescence [219].

#### 3.2.1. RIPK1

RIPK1 is more prone to undergoing autophosphorylation to stimulate downstream signaling, possibly because of its own weaker kinase activity [125]. RIPK1 S166 autophosphorylation has been extensively and widely studied and is well known [125,126]. In addition to S166, autophosphorylation can also occur in human S14, S15, S20, and S161 and mouse S14, S15, S161, and T169 [127]. S166 autophosphorylation may not cause cell death alone but enhances RIPK1 kinase activity, promotes autophosphorylation at other sites, and may serve as a reliable biomarker of RIPK1 kinase-dependent cell death [126]. The role regarding the phosphorylation of RIPK1 S161 is not as consistent as that of S166. It has been suggested that S161 phosphorylation has little effect on RIPK1 kinase activity [129]. In contrast, it has also been suggested that the autophosphorylation that occurs on S161 recruits more RIPK3 and therefore promotes necrosome formation and necroptosis [94]. Thus, it is not difficult to see that autophosphorylation is an indispensable factor for RIPK1 to act as a cell fate determinant.

In addition to autophosphorylation, there are many other sites where phosphorylation can affect RIPK1. In Complex I, the IKK complex phosphorylates RIPK1 and protects cells from RIPK1 kinase-dependent death [220]. A follow-up study found that RIPK1 S25 appears to be the site of action of the IKK complex [127]. Given that S25 lies within the kinase structural domain of RIPK1, phosphorylation of RIPK1 at S25 by IKKα/β directly curbs RIPK1 kinase activity and S166 autophosphorylation. This action also averts TNF-mediated, RIPK1 kinase-dependent cell death [127]. The IKK complex-mediated phosphorylation of RIPK1 S25 also promotes T cell survival [221]. MAPKAPK2 has been shown to be a key kinase in limiting RIPK1 kinase activity and inhibiting Complex I to Complex II conversion [49,50,51]. Human S320 and mouse S321 and S336 are sites where MK2 acts on RIPK1 [49,50,51]. MK2 phosphorylates RIPK1 directly at S321. This phosphorylation inhibits RIPK1’s binding to FADD/caspase-8 and its induction of RIPK1 kinase-dependent apoptosis and necroptosis [50]. The tyrosine kinases JAK1 and SRC mediate tyrosine phosphorylation of RIPK1 at Y383 (Y384 in humans). This phosphorylation inhibits RIPK1 kinase activity. Y383 tyrosine phosphorylation is crucial for MK2 binding to RIPK1 and for MK2’s further activation, though the precise mechanism remains unclear [52]. TAK1, recruited into Complex I, phosphorylates S321 in the RIPK1 intermediate domain. This phosphorylation inhibits RIPK1’s interaction with FADD/caspase-8 and suppresses cell death [128]. Similarly, the autophagy-initiating kinase ULK1 phosphorylates S357 within the RIPK1 intermediate domain, TBK1 in Complex I phosphorylates RIPK1 T147 or T189/190 (human/mouse), and AMPK phosphorylates RIPK1 S415, which can inhibit RIPK1-dependent death [53,54,55].

Interestingly, RIPK1 S89 phosphorylation restricts its kinase activity, and the RIPK1 S89A mutation up-regulates RIPK1 activity, but the RIPK1 S89D mutation down-regulates RIPK1 activity, an interesting phenomenon [129].

ROS are also involved in the phosphorylation of RIPK1, which was found to accumulate in mitochondria after ROS stimulation and to undergo S166 and S321 phosphorylation, accompanied by ubiquitination. However, how this phosphorylation occurs needs to be further investigated [222].

Of course, dephosphorylation may also regulate RIPK1, but this has not been extensively studied. Protein phosphatase 1 regulatory subunit 3G (PPP1R3G) is involved in a key process. Its catalytic subunit, protein phosphatase 1 γ (PP1γ), is recruited to Complex I. There, it removes the inhibitory phosphorylation of RIPK1 at S25. This action promotes RIPK1-dependent cell death [130].

Protein 2 containing SET and MYND structural domains (SMYD2) catalyzes the lysine methylation of histones and non-histone proteins, but new studies have found that SMYD2 can regulate the phosphorylation of RIPK1 and thus inhibit necroptosis; however, the exact phosphorylation site is unknown [131] (Figure 1A, Figure 2A,B and Table 1).

#### 3.2.2. RIPK3

RIPK3 has a similar structure to RIPK1, but without the DD domain. The difference is that TRIF and ZBP1 can interact with RIPK3 alone without RIPK1. During the activation of RIPK3, RIPK1 acts more as a scaffold rather than as its upstream kinase [85,223]. As with RIPK1, we first discuss the autophosphorylation of RIPK3. The following autophosphorylation sites have been identified, including S199, S211, S215, S227, and T182 in human RIPK3 and S204, S232, and T231 in mouse RIPK3. RIPK3 S227 is the key site for its interaction with MLKL [147,148,149,150]. The phosphorylation of human RIPK3 at T224 and S227 acts synergistically to boost its interaction with MLKL. Under normal resting conditions, the autophosphorylation of RIPK3 at S227 depends on MLKL. However, during necroptosis, this autophosphorylation of S227 becomes independent of MLKL [150]. T224 is not conserved in vivo in mice, possibly implying species-specific differences in RIPK3-MLKL interactions. Similarly, mouse T231/S232 and human S227 are critical for recognition of their homologous MLKL immediate homologs, which is also species-specific [171]. RIPK3 S232 also mediates necroptosis-induced periodontitis in mice [224]. RIPK3 S227 can not only undergo autophosphorylation but can also be modified by other kinases. CK1α, CK1δ, and CK1ε in the casein kinase 1 (CK1) family can bind to RIPK3 during necrosome formation, phosphorylate S227, and further activate necroptosis [153]. However, additional studies have shown that the CK1 family member casein kinase 1G2 (CSNK1G2) inhibits the activation of RIPK3 dimerization and inhibits necroptosis by facilitating the binding of RIPK3 to RIPK3 monomers through RIPK3 S211/T215 autophosphorylation [151]. Thus, the effect of the CK1 family on RIPK3 and necroptosis is not absolute. Next, we discuss other phosphorylation sites. T182 is thought to be the initiating event for S227 autophosphorylation, and the substitution of T182 by alanine (T182A) abrogates S227 phosphorylation and prevents TNFα-induced necroptosis. Interestingly, when RIPK3 is phosphorylated at T182, it interacts with the forkhead-associated (FHA) domain of PELI1. This interaction leads to the PELI1-mediated polyubiquitination of RIPK3 at K363 with K48-linkages. Subsequently, RIPK3 undergoes proteasomal cleavage, thereby inhibiting necroptosis [143]. Substituting S204 (S199A in human RIPK3 and S204A in mouse RIPK3) abolishes the in vitro kinase activity of RIPK3. In contrast, aspartate-substitution mutants (S204D in mouse RIPK3 and S199D in human RIPK3) maintain this in vitro activity. In necroptosis, RIPK1’s key role is to promote the phosphorylation of RIPK3 at S204, either directly or indirectly [129].

The phosphorylation of RIPK3 also mediates a switch to apoptosis. When the levels of the RIPK3 chaperone heat shock protein 90 (Hsp90) and its co-chaperone Cdc37 (CDC37) are low, RIPK3 gets phosphorylated at the S165/T166 site. This phosphorylation inactivates RIPK3 kinase activity and its capacity to recruit RIPK1, FADD, and caspase-8 for forming a cytoplasmic caspase-activated complex. As a result, apoptosis occurs without necroptosis [152].

Under specific conditions, Complex IIb can assemble into structures called ripoptosomes. In the G2/M phase, RIPK3 is considered an extra component of the ripoptosome. Procaspase-8 within the ripoptosome can cleave RIPK3. Polo-like kinase 1 (PLK1) directly binds to RIPK3 and phosphorylates it at S369. This phosphorylation blocks the ripoptosome-mediated cleavage of RIPK3, preserving its pro-death activity. As a result, when mitotic errors happen, an alternative cell death pathway is available [225].

Dephosphatases also regulate RIPK3 activity. Protein phosphatase 1B (Ppm1b) prevents RIPK3 auto-activation and negatively regulates TNF-induced necrotic apoptosis in quiescent cells and is not dependent on the NF-κB pathway. Possible sites of action are T231 and S232, which can undergo autophosphorylation [154] (Figure 1A and Figure 2A,B and Table 2).

#### 3.2.3. MLKL

MLKL, a pseudokinase, lacks the catalytic activity for phosphoryl transfer seen in RIPK1 and RIPK3. This is due to the loss of two key catalytic residues related to phosphoryl transfer. One is from the catalytic loop’s “His-Arg-Asp (HRD)” motif, and the other is from the metal-cofactor binding “Asp-Phe-Gly (DFG)” motif [165].

There seems to be a consensus that MLKL, being downstream of RIPK3, is phosphorylated by it and thus leads to cell death. Phosphorylated MLKL oligomerizes and binds to phosphatidylinositol lipids and cardiolipin. This enables its translocation from the cytoplasm to the plasma and intracellular membranes, where it directly impairs membrane integrity, triggering necroptosis [172]. During this, RIPK3 can phosphorylate T357 and S358 in human MLKL and S345 in mouse MLKL. The phosphorylation of T357 and S358 “opens” the folded MLKL structure, linking the 4HB domain to the C-terminal pseudokinase domain. Following this, phosphorylated MLKL undergoes a conformational change that aids oligomerization [165,171,172]. Calcium/calmodulin-dependent protein kinase II (CAMK2/CaMKII), rather than RIPK3, phosphorylates MLKL at the same site and can promote autophagy [173]. Interestingly, both pharmacological and alcoholic liver injury were accompanied by MLKL aggregation and S358 phosphorylation, suggesting that MLKL S358 phosphorylation may be one of the biomarkers of liver injury [172,226].

In addition to S345, mouse S158, S228, S248, S347, and T349 can be phosphorylated by RIPK3 but have different functions [174,175]. S347 has an auxiliary role to S345, as additional mutations in this residue render MLKL completely ineffective against necroptosis. Phosphorylation of S158, S228, and S248 also regulates necroptosis, and the function of the phosphorylation of T349 is unknown [174,175]. MLKL S441 phosphorylation promotes myelin degradation [176].

In addition to RIPK3, receptor tyrosine kinases from the TAM (Tyro3, Axl, and Mer) family phosphorylate MLKL at Y376. This phosphorylation controls MLKL oligomerization, not its membrane translocation or RIPK3 phosphorylation, and promotes necroptosis [177].

Phosphorylation not only promotes cell death but also inhibits necroptosis and autoinflammation. In humans, phosphorylation of MLKL at S83, or in mice at S82, curbs MLKL activity following RIPK3-mediated MLKL activation [178]. This indicates that MLKL phosphorylation has a dual-sided role in regulating necroptosis (Figure 1A and Figure 2A,B and Table 3).

#### 3.2.4. FADD

FADD was first reported to have two phosphorylated forms called CAP1 and CAP2 [227]. FADD can be phosphorylated at S194 (phosphorylated by casein kinase I alpha (CKIα)) [187,188], S200 (phosphorylated by the anti-apoptotic kinase CK2) [189], S203 (phosphorylated by the mitotic kinases Aurora-A (Aur-A) and PLK1) [190] and is associated with cellular sublocalization. It follows that FADD phosphorylation is less closely associated with cell death (Table 4).

#### 3.2.5. Caspase-8

Phosphorylation of Procaspase-8 T265 is essential for the promotion of necroptosis [228]. Ribosomal protein S6 kinase A1 (p90 RSK) can be activated by 3-phosphatidylinositol-dependent protein kinase 1 (PDK 1) through a non-classical mechanism. Subsequently, p90 RSK phosphorylates procaspase-8 T265. The phosphorylation of procaspase-8 T265 maintains necrosome integrity [196].

There are many other phosphorylation sites on caspase-8, but they have been shown to be more associated with apoptosis, e.g., Y380, Y448, S347, S387, T263, and T273 [197,199,200,201,202,229,230,231,232] (Table 4).

#### 3.2.6. cFLIP

To date, three cFLIP phosphorylation sites have been identified: T166, S193, and S273. Interestingly, T166 and S193 mediate the regulatory crosstalk between cFLIP phosphorylation and ubiquitination. cFLIP_L_ T166 has been shown to be required for K167 ubiquitination [208,209], thereby signaling to promote proteasomal degradation. In contrast, phosphorylation of protein kinase C (PKC) at S193 blocked the ubiquitination of the c-FLIPS and c-FLIPL isoforms at K195 and K192 and inhibited their degradation [207]. In addition, TNFα stimulates the AKT serine/threonine kinase 1 (Akt1)-mediated phosphorylation of cFLIP_L_ S273, thereby promoting the proteasomal degradation of cFLIP_L_ [212]. The degradation of cFLIPL has a negative effect on the inhibition of caspase-8 activity, and its propagation of death signaling requires further validation.

### 3.3. Glycosylation

Protein glycosylation can be classified into O-linked and N-linked glycosylation, depending on how glycosidic bonds are formed. O-Glc*NA*cylation, catalyzed by O-Glc*NA*c transferase (OGT) and reversed by O-Glc*NA*case (OGA), plays a role in regulating processes such as host immune responses and signal transduction during pathogen infection. *O*-Glc*NA*cylation negatively regulates necroptosis. Upon necroptosis stimulation of erythrocytes, the *O*-Glc*NA*cylation of RIPK1 S331 is decreased and the phosphorylation of RIPK1 S166 is enhanced, promoting the formation of the necrosome. The OGA inhibitor Thiamet-G (TMG) reverses the decrease in the *O*-Glc*NA*cylation of RIPK1 and promotes necroptosis [132] (Figure 1A, Figure 2A,B and Table 1). The product of the hexosamine biosynthetic pathway (HBP), O-conjugated β-N-acetylglucosamine (*O*-Glc*NA*c), modifies RIPK3 via OGT. *O*-Glc*NA*cylation of RIPK3 on T467 inhibits its RHIM function and therefore inhibits necroptosis [155]. Sevoflurane (SEVO) increases the *O*-Glc*NA*cylation of RIPK3 and inhibits RIPK3 binding to MLKL, inhibiting necroptosis induced by myocardial ischemia–reperfusion injury (MIRI) [156]. OGT glycosylates RIPK3 and reduces the stability of the RIPK3 protein and therefore inhibits necroptosis, so that the loss of *O*-Glc*NA*c leads to liver fibrosis and inflammation due to unregulated necroptosis [157]. In the brains of Alzheimer’s disease (AD) patients, elevated O-GlcNAcylation of RIPK3 hampers RIPK3 phosphorylation and its interaction with RIPK1. This leads to a reduction in necroptosis, potentially offering protection against AD [158]. The traditional Chinese medicine Wu-Mei-Wan (WMW) alleviates colitis in mice. It does so by inhibiting necroptosis through enhancing RIPK3 O-Glc*NA*cylation [159] (Figure 1A and Figure 2A,B and Table 2).

*Enteropathogenic Escherichia coli* (EPEC) and *Salmonella typhimurium* (*S. typhimurium*) possess a type III secretion system (T3SS) with effectors NleB and SseK1/2/3, respectively. NleB and SseK1/2/3 can modify a number of DD proteins by Arg-Glc*NA*cylation, such as TRADD, FADD, and RIPK1. NleB can modify TRADD-DD R235 and RIPK1-DD R603, and SseK1/2/3 can modify TNFR1-DD R376, TRADD-DD R233/235/245, and TRAILR-DD R293/359 [133,183,184,185,233,234,235]. This blocks their signaling and prevents necroptosis from occurring (Table 1 and Table 4).

### 3.4. Methylation

Methylation acts through methyltransferases, demethylases, and methylation-dependent binding proteins, which are three methylation-related enzymes that perform writing, erasing, and recognition functions, respectively [236]. Methylation can occur at the DNA level, the RNA level, and the protein level, which are catalyzed by different enzymes. Protein-level methylation can be categorized into histone and non-histone methylation. Arginine and lysine are common amino acids subject to protein methylation. Arginine methylation is mediated by proteins from the protein arginine methyltransferase (PRMT) family, while lysine methylation is carried out by lysine methyltransferase (KMT) [237]. PRMT1 can methylate human RIPK3 R486 and mouse RIPK3 R479, thereby inhibiting RIPK1-RIPK3 interactions and RIPK3 phosphorylation, respectively, and suppressing necrosome formation [160]. Both PRMT1 and PRMT5 interact with RIPK1. They mediate symmetric arginine dimethylation of R486 at the C-terminus of human RIPK3. The methylation of RIPK3 inhibits necroptosis. It does this by blocking RIPK1 S166 autophosphorylation and suppressing downstream signaling [161] (Figure 1A, Figure 2A,B, and Table 2). TRAF2, which forms Complex I, can be methylated by SMYD2, promoting NF-κB expression and thus inflammatory signaling [238].

### 3.5. Acetylation

Acetylation involves adding acetyl groups, sourced from acetyl coenzyme A (acetyl-CoA), to specific residues in proteins [239]. Acetyltransferases, such as histone acetyltransferases (HATs), lysine acetyltransferases (KATs), and Nα-acetyltransferases (NATs), are responsible for adding these acetyl groups. Conversely, deacetylases, including histone deacetylases (HDACs) and NAD^+^-dependent sirtuins (SIRTs), remove them [240]. Protein acetylation is divided into histone acetylation and non-histone acetylation. It was shown that RIPK1 can form a complex with HAT1 and SIRT1. RIPK1 can be acetylated in the kinase active region at K115; in the DD region at K625, K627, K642, and K648; and next to the DD region at K596 and K599. Cellular RIPK1-caspase-8-dependent apoptosis was enhanced by the use of SIRT1 inhibitors, suggesting that RIPK1 may promote apoptosis upon acetylation, thus affecting the biological behavior of tumor cells and influencing tumor development [134]. It is reasonable to speculate whether RIPK1 acetylation may also promote necroptosis when caspase-8 activity is inhibited (Figure 1A, Figure 2A,B and Table 1). Conversely, STIR2 can bind RIPK3. When TNF-α is stimulated, RIPK1 is activated and binds more easily to the STIR2-RIPK3 complex. This complex then deacetylates RIPK1. The likely acetylation site of RIPK1 is near K530 in the RHIM structural domain. This deacetylation process promotes the start of necroptosis (Figure 1A, Figure 2A,B, and Table 2). In acute oxalate nephropathy, inhibition of HDAC6 reduces necroptosis. In this process, HDAC6 does not appear to act directly with proteins such as RIPK1 and RIPK3 to exert its deacetylation effect, but rather, with microtubule proteins to promote inflammatory signaling, and the exact mechanism needs to be further elucidated [241]. HDAC3 indirectly regulates necrotic apoptosis. Macrophages deficient in HDAC3 have elevated histone acetylation and increased cathepsin B (CTSB), leading to increased degradation of RIPK1 and inhibition of inflammatory signaling or death signaling [242].

### 3.6. Disulfide Bonds

The formation and breaking of disulfide bonds can also be considered as a type of PTM. ROS promotes the formation of disulfide bonds between RIPK1 C257, C268, and C586; promotes RIPK1 S161 autophosphorylation; and increases the affinity for RIPK3, enhancing death signaling [94] (Figure 1A and Figure 2A,B and Table 1). Hypothiocyanic acid (HOSCN) acts to inhibit caspase-8 activity and promote necroptosis. It does this by catalyzing the formation of a disulfide bond. This bond links dimers between C360 in the large catalytic subunit and C409 in the small catalytic subunit of caspase-8 [243] (Table 4). MLKL forms disulfide-dependent amyloid polymers via disulfide bonds. This might occur because RIPK1/3 polymers phosphorylate MLKL, causing MLKL molecules to be spaced apart. Then, intramolecular and intermolecular disulfide bonds form through cysteines. This further promotes MLKL tetramer formation and the start of necroptosis. Inhibiting disulfide bond formation at human MLKL C86 can block MLKL polymer formation and subsequent cell death [179]. The thiol oxidoreductase thioredoxin-1 (Trx1) has C32 that cross-links with C86 of human MLKL. This keeps MLKL in a reduced state, inhibits its disulfide bonding, and blocks MLKL polymer formation. Thus, Trx1 can be regarded as an inhibitor of necroptosis [180] (Figure 1A and Figure 2A,B, and Table 3).

### 3.7. Caspase Cleavage

Protein cleavage mediated by the caspase family can lead to changes in protein structure and is also a type of PTM. Caspase-8 can be inhibited by the cleavage of RIPK1 D324 and RIPK3 D328 [135,162] (Table 1 and Table 2). Not only can caspase-8 cleave RIPK1, but so can caspase-6 and caspase-10, and the cleavage sites appear to be consistent [135]. RIPK1 can be activated in a DNA double-strand break (DSB)-induced signaling platform called the ripoptosome. When caspase is active, caspase-6-mediated cleavage inactivates RIPK1 completely, resulting in apoptosis. However, when caspase is inactive, double-strand breaks (DSBs) enhance NF-κB signaling and the generation of pro-inflammatory cytokines. If caspase-8 is not activated simultaneously, TNF-α signaling stimulation promotes necroptosis [136]. In addition to its cysteine enzyme activity, caspase-6 promotes necroptosis through binding directly to RIPK3. The binding of RIPK3 to the RHIM of ZBP1 to promote necroptosis is not required for its caspase activity [244]. In primary biliary cholangitis (PBC), macrophage caspase-10 has a greater cleavage capacity than caspase-8, with an increased ability to form complexes with RIPK1 and FADD, which can better cleave RIPK1 and inhibit necroptosis, and *caspase-10* knockout macrophages are more likely to trigger pyroptosis and necroptosis [137] (Figure 1A and Figure 2A,B).

### 3.8. Nitrosylation

In cerebral ischemia–reperfusion, inward calcium currents mediated by NMDA receptors activate neuronal nitric oxide synthase, which in turn induces NO production. The C119 residue of RIPK3, when nitrosylated by NO S-nitrosylation, strengthens its kinase activity and contributes to the development of apoptosis and necroptosis [163,164] (Table 2). Caspase-8 can be S-nitrosylated by nitric oxide, which inhibits its activity and interrupts its apoptotic signaling, and whether it promotes necroptosis signaling and its specific modification sites need to be further investigated [203]. The nitrosylation of cFLIPL at C254 and C259 blocks its ubiquitination and proteasomal degradation. This leads to an increase in the cFLIPL concentration. Ordinarily, elevated cFLIPL would promote the formation of the cFLIPL-caspase-8 heterodimer, enhancing caspase-8 activity. However, nitrosylation of the cFLIPL p20 subunit weakens its capacity to stabilize the active site of the cFLIPL-caspase-8 heterodimer. As a result, it functions as an inhibitor of caspase-8 activity [213] (Figure 1A and Figure 2A,B, and Table 4).

### 3.9. SUMOylation

The small ubiquitin-like modifier (SUMO) can be attached to the FADD DD at K120, K125, and K149 by the E3 SUMO protein ligase PIAS3. This SUMOylation may be linked to a form of mitochondrial fission and caspase-10-related cell death [191]. Caspase-8 can be SUMOylated at K156. This modification is associated with the nuclear localization of caspase-8 but does not impact its activation [204] (Table 4). RIPK1 is SUMOylated at K550, and the SUMOylation of RIPK1 promotes its activation, which is reversed by SUMO-specific protease 1 (SENP1), inhibiting cell death and the development of non-alcoholic steatohepatitis [138] (Figure 1A and Figure 2A,B and Table 1).

## 4. Conclusions

Necroptosis activation stems from diverse stimuli and undergoes regulation via a myriad of signaling mediators and their post-translational modifications. These elements form sophisticated interdependent networks that fundamentally modulate necroptotic progression. Beyond the aforementioned molecular components, numerous undiscovered entities pertinent to necroptotic pathways warrant comprehensive investigation in forthcoming research endeavors. FK506-binding protein 12 (FKBP12) regulates protein folding and conformational changes. It is closely linked to the autophosphorylation of RIPK1 and RIPK3, as well as the formation of the necrosome [245]. ZFP91 induces the deubiquitination of RIPK1 to stabilize RIPK1 to promote cell death, promotes RIPK1-RIPK3 interactions to stabilize RIPK1 and RIPK3 proteins, and promotes necrotic apoptosis but also promotes mitochondrial ROS production [68]. Under normoglycemia, Cannabinoid receptor 2 (CB2R) represses the expression of RIPK1, RIPK3, and MLKL at the transcriptional level. However, under hyperglycemia, MLKL phosphorylates CB2R to mediate its ubiquitination degradation and promote necroptosis and diabetic heart dysfunction [246]. Interferon-inducible 2′-5′ oligoadenylate synthetase-like (OASL) provides a scaffold for ZBP1-RIPK3-MLKL binding in virus-induced necroptosis and thus has potent antiviral activity [247]. Molecules such as these are increasingly being discovered and studied, suggesting that necroptosis has additional undiscovered mechanisms.

Necroptosis is linked to numerous human diseases. For instance, in infectious diseases such as various viral and bacterial infections, it is associated with the activation of TLRs or ZBP1 [248,249]; it is also associated with tissue injury, including the quantity and type of ischemia–reperfusion injury [250]; organ damage such as liver, pulmonary, myocardial and interstitial fibrosis [251,252]; cardiovascular diseases such as atherosclerosis; central nervous system diseases; liver diseases such as chronic inflammation and fibrosis in aging liver [251,253]; intestinal diseases such as inflammatory bowel diseases; and autoimmune diseases, all of which are associated with human inflammatory diseases [254]. Moreover, necroptosis and its post-translational modifications (PTMs) play crucial roles in tumor development and treatment. This has significant implications for understanding oncological pathophysiological mechanisms and advancing therapeutic research [255,256]. Therefore, by gaining a deep understanding of the mechanisms of necroptosis and the means of its PTMs, we can use a wide range of agonists and inhibitors to promote or prevent the onset of necroptosis and to act as a therapeutic treatment for disease. For instance, different kinase inhibitors can be used to block the kinase activity of RIPK1. Ubiquitination agonists can also be applied to modify RIPK1 further and inhibit necroptosis. This may prevent, reduce, or delay the start and development of some diseases. Necroptosis inhibitors such as Nec-1s and GSK’872 can effectively suppress the expression of p-MLKL. As a result, they significantly inhibit fat necrosis and subsequent fibrosis in fat grafts [257]. In addition, the RIPK1 inhibitor GSK2982772 (compound 5) has the potential to become an effective treatment for psoriasis, rheumatoid arthritis, and ulcerative colitis [258]. In addition, epigenetic silencing of RIPK3 in hepatocytes is a potential target for novel drugs that have been shown to inhibit MLKL-mediated necroptosis, which induces various liver pathologies [259]. There are a number of other drugs that can exert a therapeutic effect by inhibiting necroptosis, such as ursolic acid in intestinal ischemia–reperfusion injury via STAT3 signaling [260] and rapamycin in doxorubicin-induced cardiomyopathy via the RIPK3-MLKL pathway, whose PTMs need to be explored and identified [261]. Conversely, drugs and therapies designed to promote tissue necroptosis by regulating its PTMs can be used to treat several cancers. For example, a combination of SHK and Chi-Ag NPs is able to induce effective ICD in triple-negative breast cancer tissues by synergistically inducing tumor cell necroptosis through the upregulation of RIPK3, pRIPK3, and tetrameric MLKL expression [262]. The novel isobavachalcone (compound 16) also has a therapeutic effect in NSCLC by upregulating RIPK3 and MLKL to mediate necroptosis [263]. The novel small molecule VDX-111 induces necroptosis. It does so by regulating RIPK1 expression, thereby inhibiting the progression of ovarian cancer [264]. Table 5 summarizes part of these agents, including well-characterized inhibitors such as GSK’872 (RIPK3) and GSK2982772 (RIPK1), as well as emerging candidates under preclinical and clinical evaluation. This table serves as a valuable resource for understanding the therapeutic landscape of necroptosis-targeting drugs and their potential applications in disease management.

In addition to the targeted agents mentioned above, for which specific effects are known, there are other potential targets for further investigation. Glycosylation has been shown to modify proteins on RIPK1, RIPK3, and the DISC complex in necroptosis, and all appear to negatively regulate them; for example, the *O*-Glc*NA*cylation of human RIPK1 S331 can inhibit the formation of the RIPK1-RIPK3 complex [132], OGT mediates *O*-Glc*NA*cylation on RIPK3 T467, which can inhibit necroptosis to treat septic inflammation [155], and glycosylation has been linked to metabolism as well [133,155,157,158,159,183,184,185,233,234]. This may be a trend for future research. The effects of methylation and acetylation on the modulation of necroptosis are unambiguous and may therefore also be targets for drug design [134,160,161]. Additionally, phosphorylation also plays a significant role in the regulation of necroptosis, and proteins such as IKKα/β [220], TBK1 [54], MK2 [50], Pellino 1 [143], CK1 [272], and TAM [177] may be targets for novel drugs. Dephosphorylated proteins such as Ppm1b [154] and TRAF2 [57] have also been implicated in necroptosis. In addition, more kinases and ubiquitination E3 ligases have been identified, such as JAK1, SRC [52], ULK1 [53], PARdU [119], MG53 [120], CHIP [123], cIAP1/cIAP2 [113], LUBAC [115,116], MIB2 [118], A20, CYLD, OTULIN [66,273], Pellino 1, c-Cbl [121,122], and so on, which could be the targets for pharmacological intervention. An increasing number of modification sites are also being found on molecules such as RIPK1, RIPK3, and MLKL, which merit further investigation by researchers. This may drive the development of new targeted drugs to augment this mechanism. Such drugs could potentially delay or treat a broad spectrum of diseases. These include inflammatory, injury-related, immunological, or oncological conditions such as psoriasis, severe rheumatoid arthritis, and Alzheimer’s disease [41].

Secondly, we need to discover more means of PTMs, more modification sites, and their crosstalk in necroptosis. For example, it may be possible to correlate necroptosis with other PTMs, such as neddylation, palmitoylation, and lactylation, which is now a hot research topic. With the continuous development of protein resolution techniques and mass spectrometry, we can easily resolve the structure of the molecules in necroptosis and their PTM sites, so more modification sites are worth discovering and verifying. Interestingly, different PTMs can exist at the same site on the same molecule, for example, RIPK1 K115 is modified by both the E3 ligase PELI1 and the methylation-associated enzymes SIRT1, SIRT2, HAT1, and HAT4 [121,134], and we cannot help but wonder if the two PTMs compete with each other. If so, which PTM dominates during necroptosis? There are also complex relationships between different PTMs at different sites on the same molecule or between different molecules. For example, the *O*-Glc*NA*cylation of RIPK1 S331 promotes autophosphorylation of RIPK1 S166 and promotes necroptosis [132]. Disulfide bond formation of RIPK1 C257, C268, and C586 promotes RIPK1 S161 autophosphorylation and enhances its kinase activity [94]. PRMT5 exerts its methylation by interacting directly with RIPK1 and reducing its physical distance from RIPK3 [161]. In addition, we can not only look at PTMs but can also study more at the level of DNA and RNA regulation, which also allows us to understand the mechanism of their occurrence. There is an abundance of studies that have indicated that lncRNAs play an essential role in the initiation and development of necroptosis through protein–protein interactions, transcriptional control, and PTMs undoubtedly. lncRNAs have complicated roles in the regulation of necroptosis pathways, and in the treatment of cancer by necroptosis, the use of lncRNA-based drug technologies is expected to inhibit the progression, spread, and metastasis of cancer by regulating necroptosis [274,275].

Finally, we can explore whether crosstalk is possible with other modes of death via PTMs, such as cellular pyroptosis, ferroptosis, autophagy, pan-apoptosis, etc. Termination of one type of death under certain conditions will result in the transformation of another type of death, and by studying the key molecules involved and their PTMs, this will also facilitate our development of clinical drug targets. For example, phosphorylation of the necroptosis-related pathway molecules RIPK1, RIPK3, and MLKL is increased in acidic pH environments, inhibiting necroptosis and promoting its conversion to apoptosis [276]. Moreover, cell death pathways including apoptosis, necroptosis, pyroptosis, and ferroptosis can have continuous and interconnected roles in specific pathologies and pathophysiological processes. These pathways may be regulated by post-translational modifications (PTMs) [3]. All of these questions concerning necroptosis and its PTMs deserve a considerable amount of further research and demonstration.

## Figures and Tables

**Table 1:** PTMs of RIPK1.

Species	Site	Domain	Protein	Necroptosis	References
Ubiquitination					
Human	K377	Intermediate	cIAP1, cIAP2	↓	[42] [114]
Mouse	K376	Intermediate	cIAP1, cIAP2	↓	[42] [114]
Human and Mouse	Unknown	Unknown	cIAP1(UBA domain)	↓	[113]
Human and Mouse	Unknown	Unknown	LUBAC	↓	[44] [45] [46] [47]
Human	K377	Intermediate	MIB2	↓	[118]
Human	K634	Death domain	MIB2	↓	[118]
Mouse	K376	Intermediate	PARdU	↓	[119]
Human	K377	Intermediate	PARdU	↓	[119]
Human	K316	Intermediate	MG53	↓	[120]
Human	K604, K627	Death domain	MG53	↓	[120]
Human	K377	Intermediate	A20, OTULIN CYLD, USP21,	↑	[62] [63] [64] [65] [66] [67]
Mouse	K376	Intermediate	A20, OTULIN CYLD, USP21,	↑	[62] [63] [64] [65] [66] [67]
Human and Mouse	Unknown	Unknown	ZFP91	↑	[68]
Human	K115	Kinase domain	PELI1	↑	[121]
Mouse	K115	Kinase domain	PELI1	↑	[121]
Human	K158	Kinase domain	c-Cbl	↑	[122]
Human	K571	Intermediate	CHIP	↓	[123]
Human	K604, K627	Death domain	CHIP	↓	[123]
Mouse	K612	Death domain	Unknown	↓	[124]
Human	K13	N-terminal (prior to Kinase domain)	Unknown	Unknown	[124]
Human	K302, K305, K306, K396, K530, K565, K585	Intermediate	Unknown	Unknown	[124]
Human	K30, K45, K49, K65, K77, K97, K105, K132, K137, K140, K153, K163, K167, K184, K185, K204, K265, K284	Kinase domain	Unknown	Unknown	[124]
Human	K596, K599, K642, K648	Death domain	Unknown	Unknown	[124]
Mouse	K13, K20	N-terminal (prior to Kinase domain)	Unknown	Unknown	[124]
Mouse	K306, K307, K392, K395, K429, K519, K550, K584	Intermediate	Unknown	Unknown	[124]
Mouse	K30, K45, K46, K65, K77, K105, K137, K140, K153, K163, K167, K205	Kinase domain	Unknown	Unknown	[124]
Mouse	K589, K619, K627, K633	Death domain	Unknown	Unknown	[124]
Phosphorylation					
Human	S166	Kinase domain	RIPK1	↑	[125] [126]
Human	S161	Kinase domain	RIPK1	↑	[94]
Human	S14, S15, S20	N-terminal (prior to Kinase domain)	RIPK1	No effect	[127]
Mouse	S14, S15	N-terminal (prior to Kinase domain)	RIPK1	No effect	[127]
Mouse	T169	Kinase domain	RIPK1	Unknown	[127]
Human	S25	Kinase domain	IKKα/β	↓	[127]
Mouse	S25	Kinase domain	IKKα/β	↓	[127]
Human	S320	Intermediate	MK2	↓	[49] [50] [51]
Mouse	S321, S336	Intermediate	MK2	↓	[49] [50] [51]
Mouse	S321	Intermediate	TAK1	↓	[128]
Human	Y384	Intermediate	JAK1, SRC	↓	[52]
Mouse	Y383	Intermediate	JAK1, SRC	↓	[52]
Human	S357	Intermediate	ULK1	↓	[53]
Human	T147	Kinase domain	TBK1	↓	[54]
Mouse	S189, S190	Kinase domain	TBK1	↓	[54]
Human	S416	Intermediate	AMPK	↓	[55]
Mouse	S415	Intermediate	AMPK	↓	[55]
Human	S89	Kinase domain	Unknown	↓	[129]
Mouse, rat, xenopus, zebrafish	S89	Kinase domain	Unknown	↓	[129]
Human	S25	Kinase domain	PPP1R3G	↑	[130]
Mouse	S25	Kinase domain	PPP1R3G	↑	[130]
Human	Unknown	Unknown	SMYD2	↓	[131]
Glycosylation					
Human	S331	Intermediate	OGT	↓	[132]
Mouse	S332	Intermediate	OGT	↓	[132]
Human	R603	Death domain	NIeB2	↓	[133]
Acetylation					
Human	K115	Kinase domain	SIRT1, SIRT2	↓	[134]
Human	K115	Kinase domain	HAT1, HAT4	↑	[134]
Human	K625, K627, K642, K648	Death domain	SIRT1, SIRT2	↓	[134]
Human	K625, K627, K642, K648	Death domain	HAT1, HAT4	↑	[134]
Human	K596, K599	Intermediate	SIRT1, SIRT2	↓	[134]
Human	K596, K599	Intermediate	HAT1, HAT4	↑	[134]
Disulfide bonds					
Human	C257, C268	Kinase domain	ROS	↑	[94]
Human	C586	Intermediate	ROS	↑	[94]
Caspase cleavage					
Human	D324	Intermediate	Caspase-6, -8, -10	↓	[135] [136] [137]
Mouse	D325	Intermediate	Caspase-6, -8, -10	↓	[135] [136] [137]
SUMOylation					
Human	K550	Intermediate	SENP1	↓	[138]

Downward-pointing arrows mean that necroptosis is inhibited, and upward-pointing arrows mean that necroptosis is promoted.

**Table 2:** PTMs of RIPK3.

Species	Site	Domain	Protein	Necroptosis	References
Ubiquitination					
Human and Mouse	K5	N-terminal (prior to Kinase domain)	A20	↓	[139] [140]
Mouse	K158, K287	Kinase domain	Unknown	↓	[139]
Mouse	K307	Intermediate	Unknown	↓	[139]
Mouse	K469	C-terminal (after RHIM domain)	Unknown	↓	[139]
Mouse	K359	Intermediate	Unknown	↑	[139]
Human	K42	Kinase domain	USP22	No effect	[142]
Human	K351	Intermediate	USP22	No effect	[142]
Human	K518	C-terminal (after RHIM domain)	USP22	↑	[142]
Human	K55	Kinase domain	CHIP	↓	[123]
Human	K363	Intermediate	CHIP	↓	[123]
Human	K363	Intermediate	PELI1	↓	[143]
Human	K501	C-terminal (after RHIM domain)	TRIM25	↓	[144]
Human	K264	Kinase domain	Unknown	↓	[145]
Human	K197	Kinase domain	Parkin	↓	[146]
Human	K302, K364	Intermediate	Parkin	↓	[146]
Phosphorylation					
Human	S199, S211, S215, S227, T182, T215	Kinase domain	RIPK3	↑	[147] [148] [149] [150] [151] [150]
Mouse	S204, S232, T231	Kinase domain	RIPK3	↑	[147] [148] [149] [150]
Human and Mouse	S164, T165	Kinase domain	RIPK3	↓	[152]
Human	T224	Kinase domain	Unknown	↑	[150]
Human	S227	Kinase domain	CK1α, CK1δ, CK1ε	↑	[153]
Mouse	S204	Kinase domain	RIPK1	↑	[129]
Human and Mouse	S164, T165	Kinase domain	RIPK3-Hsp90/CDC37	↓	[152]
Mouse	S369	Intermediate	PLK1	↑	[127]
Mouse	S232, T231	Kinase domain	Ppm1b	↓	[154]
Glycosylation					
Human	T467	RHIM domain	OGT	↓	[155] [156] [157] [158] [159]
Methylation					
Human	R486	C-terminal (after RHIM domain)	PRMT1, PRMT5	↓	[160] [161]
Mouse	R479	C-terminal (after RHIM domain)	PRMT1, PRMT5	↓	[160] [161]
Caspase cleavage					
Human	D328	Intermediate	Caspase-8	↓	[162]
Mouse	D328	Intermediate	Caspase-8	↓	[162]
Rat	D328	Intermediate	Caspase-8	↓	[162]
Nitrosylation					
Human	C119	Kinase domain	NO	↑	[163] [164]

Downward-pointing arrows mean that necroptosis is inhibited, and upward-pointing arrows mean that necroptosis is promoted.

**Table 3:** PTMs of MLKL.

Species	Site	Domain	Protein	Necroptosis	References
Ubiquitination					
Mouse	K51, K77	4HB	Unknown	↑	[166]
Mouse	K172	Brace	Unknown	↑	[166]
Mouse	K219	Pseudokinase	Unknown	↑	[166]
Human	K230	Pseudokinase	Unknown	↑	[166]
Mouse	K9, K51, K69	4HB	USP21	↑	[167]
Human	K50	4HB	ITCH	↑	[168]
Mouse	K50, K51	4HB	ITCH	↑	[168]
Human and Mouse	Unknown	Unknown	Skp2	↓	[169]
Phosphorylation					
Human	T357, S358	Pseudokinase	RIPK3	↑	[165] [171] [172]
Human	T357, S358	Pseudokinase	CAMK2/CaMKII	↑	[173]
Mouse	S345	Pseudokinase	RIPK3	↑	[165] [171] [172]
Mouse	S347	Pseudokinase	RIPK3	No effect	[174] [175]
Mouse	S158	Brace	RIPK3	↑	[174] [175]
Mouse	S228, S248	Pseudokinase	RIPK3	↑	[174] [175]
Mouse	T349	Pseudokinase	RIPK3	Unknown	[174] [175]
Mouse	S441	Pseudokinase	Unknown	Unknown	[176]
Human and Mouse	Y376	Pseudokinase	TAM	↑	[177]
Human	S83	4HB	Unknown	↓	[178]
Mouse	S82	4HB	Unknown	↓	[178]
Disulfide bonds					
Human	C86	4HB	MLKL	↑	[179]
Human	C86	4HB	Trx1	↓	[180]

Downward-pointing arrows mean that necroptosis is inhibited, and upward-pointing arrows mean that necroptosis is promoted.

**Table 4:** PTMs of TRADD, TRAILR, TNFR1, FADD, caspase-8, and cFLIP.

Species	Site	Domain	Protein	Necroptosis	References
TRADD					
Glycosylation					
Human	R235, R245	Death domain	SseK1	↓	[183] [184]
Mouse	R233	Death domain	SseK1	↓	[183]
Human	R235	Death domain	NIeB	↓	[185]
TRAILR					
Glycosylation					
Human	R359	Death domain	SseK3	↓	[183]
Mouse	R239	Death domain	SseK3	↓	[183]
TNFR1					
Glycosylation					
Human	R376	Death domain	SseK3	↓	[183]
Mouse	R376	Death domain	SseK3	↓	[183]
Ubiquitination					
Human and Mouse	Unknown	Unknown	RNF8	↑	[186]
FADD					
Ubiquitination					
Human	K149, K153	Death domain	CHIP	↓	[181]
Human and Mouse	Unknown	Unknown	MKRN1	↓	[182]
Phosphorylation					
Human	S194	Death domain	CKIα	No effect	[187] [188]
Mouse	S191	Death domain	CKIα	No effect	[187] [188]
Human	S200	Death domain	CK2	No effect	[189]
Human	S203	Death domain	Aur-A, Plk1	No effect	[190]
SUMOylation					
Human	K120, K125, K149	Death domain	PIAS3	Unknown	[191]
Caspase-8					
Ubiquitination					
Human	K461	C-terminal domain (P10 subunit)	CUL3	↓	[192]
Human and Mouse	Unknown	Unknown	TRIM13	↓	[193]
Human	K215	Intermediate	HECTD3	↓	[194]
Human	K224, K229, K231	C-terminal domain (P18 subunit)	TRAF2	Unknown	[195]
Phosphorylation					
Mouse	T265	C-terminal domain (P18 subunit)	p90 RSK	↑	[196]
Human	T263	C-terminal domain (P18 subunit)	P90 RSK (RSK2)	Unknown	[197]
Human	T273	C-terminal domain (P18 subunit)	Plk3	Unknown	[198]
Human	S347	C-terminal domain (P18 subunit)	P38-MAPK	Unknown	[199]
Human	Y380	C-terminal domain (Intermediate)	Src	Unknown	[200]
Human	S387	C-terminal domain (Intermediate)	CDK1	Unknown	[201]
Human	Y448	C-terminal domain (P10 subunit)	Lyn	Unknown	[202]
Nitrosylation					
Human and Mouse	Unknown	Unknown	NO	Unknown	[203]
SUMOylation					
Human	K156	DED domain	Unknown	Unknown	[204]
cFLIP					
Ubiquitination					
Human	K351, K353	C-terminal domain (P20 subunit)	LUBAC	↓	[205]
Human	K351, K353, K381, K386, K389, K460, K462, K473, K474	C-terminal domain	MIB2	↓	[206]
Human	K167, K192, K195	Intermediate	Unknown	Unknown	[207] [208] [209]
Human and Mouse	Unknown	Unknown	SCF^Skp2^	Unknown	[210]
Human and Mouse	Unknown	Unknown	ITCH	Unknown	[209]
Human and Mouse	Unknown	Unknown	Usp27x	Unknown	[211]
Human and Mouse	Unknown	Unknown	TRIM28	Unknown	[211]
Phosphorylation					
Human	T166	Intermediate	Unknown	Unknown	[207] [208] [209]
Human	S193	Intermediate	PKC	Unknown	[207] [208] [209]
Human	S273	C-terminal domain (P20 subunit)	Akt1	Unknown	[212]
Nitrosylation					
Human	C254, C259	C-terminal domain (P20 subunit)	NO	↑	[213]

Downward-pointing arrows mean that necroptosis is inhibited, and upward-pointing arrows mean that necroptosis is promoted.

**Table 5:** Approved and investigational drugs targeting the necroptosis pathway: targets, development stages, and associated diseases/indications.

Drug Name	Target	Development Stage	Disease/Indication	References
GSK2982772	RIPK1 inhibitor	Phase II Clinical Trial	Psoriasis, Ulcerative Colitis, Rheumatoid Arthritis	[258]
SAR443060 (DNL758)	RIPK1 inhibitor	Phase I Clinical Trial	Neurodegenerative Disorders	[265]
RIPA-56	RIPK1 inhibitor	Preclinical	Non-Alcoholic Fatty Liver Disease	[266]
Necrostatin-1s (Nec-1s)	RIPK1 inhibitor	Preclinical	Neurodegenerative Disorders, Ischemia–Reperfusion Injury, Cardiovascular Diseases, Renal Diseases, Liver Diseases, etc.	[267]
Tozasertib	RIPK1 inhibitor	Phase II Clinical Trial	Mouse Model of TNF-α-induced systemic inflammatory response syndrome	[268]
GSK3145095	RIPK1 inhibitor	Discontinued (Phase I)	Pancreatic Cancer, Colorectal Cancer	[269]
Necrosulfonamide (NSA)	MLKL inhibitor	Preclinical	Cardiac Arrest, Spinal Cord Injury, Intracerebral Hemorrhage	[270]
GSK’872	RIPK3 inhibitor	Preclinical	Ischemia–Reperfusion Injury, Sepsis, Neurodegenerative Diseases, Psoriasis, Ulcerative Colitis, Rheumatoid Arthritis	[271]
VDX-111	RIPK1 agonist	Preclinical	Ovarian Cancer	[264]
SHK+Chi-Ag NPs	RIPK3 agonist	Preclinical	Triple-Negative Breast Cancer	[262]
Seventeen isobavachalcone (IBC) derivatives (1-17)	RIPK3, MLKL agonist	Preclinical	Non-Small Cell Lung Cancer	[263]

## Data Availability

Not applicable.

## Abbreviations

4HBD	4-Helical bundle domain
ABIN	A20 binding and inhibitor of NF-kB
ABIN3	A20 binding and inhibitor of NF-kB 3
ACD	Accidental cell death
Acetyl-CoA	Acetyl coenzyme A
AD	Alzheimer’s disease
Akt1	AKT serine/threonine kinase 1
AMPK	Adenylate-activated protein kinase
AMPK	AMP-activated protein kinase
APC11	Anaphase-promoting complex subunit 11
APP	Amyloid precursor protein
Aur-A	Aurora-A
CAMK2/CaMKII	Calcium/calmodulin-dependent protein kinase II
CB2R	Cannabinoid receptor 2
CHIP	Hsp70-interacting protein
cIAP1/2	Cellular inhibitor of apoptosis 1 and 2
CK1	Casein kinase 1
CKIα	Casein kinase I alpha
CSNK1G2	Casein kinase 1G2
CTSB	Cathepsin B
CUL3	Cullin 3
CYLD	Cylindromatosis
DAI	DNA-dependent activator of IFN regulatory factors
DAMPs	Danger-associated molecular patterns
DD	Death domain
DED	Death effector domain
DFG	Asp-Phe-Gly
DISC	Death-inducing signaling complex
DR	Death receptor
DR6	Death Receptor 6
DRP1	Dynamin-related protein 1
DSB	Double-strand break
DUB	Deubiquitinating enzyme
EPEC	Enteropathogenic Escherichia coli
ER	Endoplasmic reticulum
FADD	FAS associated via death domain
FHA	Forkhead-associated
FKBP12	FK506-binding protein 12
O-GlcNAc	O-Conjugated β-N-acetylglucosamine
HATs	Histone acetyltransferases
HBP	Hexosamine biosynthetic pathway
HDACs	Histone deacetylases
HECTD3	Homologous to the E6-AP Carboxyl Terminus 3
HOIL-1L	Heme-oxidized IRP2 ubiquitin ligase-1
HOIP	HOIL-1-interacting protein
HOSCN	Hypothiocyanic acid
HRD	His-Arg-Asp
Hsp90	Heat shock protein 90
IAV	Influenza A virus
IFN-I	Interferon type I
IKK	IkB kinase
IRF1	TNF-IFN regulatory factor 1
iuRIPK1	Insoluble RIPK1 complex
K	Lysine
KATs	Lysine acetyltransferases
KMT	Lysine methyltransferase
LPS	Lipopolysaccharide
LRRK2	Leucine-rich repeat kinase 2
LUBAC	Linear ubiquitin chain assembly complex
M1	Methionine
MAPK	Mitogen-activated protein kinase
MAPKAPK2, MK2	MAPK activated protein kinase 2
MG53	Mitsugumin 53
MIB2	Mind Bomb-2
MIRI	Myocardial ischemia–reperfusion injury
MKRN1	Makorin ring finger protein 1
MLKL	Mixed lineage kinase domain-like protein
mtROS	Mitochondria ROS
MyD88	Myeloid differentiation primary response 88
NATs	Nα-acetyltransferases
NEMO	NF-κB essential modulator
OASL	Oligoadenylate synthetase-like
OGA	O-GlcNAcase
OGT	O-GlcNAc transferase
OTULIN	OTU deubiquitinase with linear linkage specificity
PARdU	PARylation-dependent ubiquitination
PARP5A	Poly(ADP-ribose) polymerase 5A
PARylation	Poly ADP-ribosylation
PBC	Primary biliary cholangitis
PDK 1	3-Phosphatidylinositol-dependent protein kinase 1
PELI1	Pellino E3 ubiquitin protein ligase 1
PGAM5	phosphoglycerate mutase family member 5
PIPs	Phosphatidylinositol phosphates
PKC	Protein kinase C
PKR	Protein kinase R
PLK1	Polo-like kinase 1
poly(I:C)	Polyinosine–polycytidylic acid
PP1γ	Protein phosphatase 1 γ
Ppm1b	Protein phosphatase 1B
PPP1R3G	Protein phosphatase 1 regulatory subunit 3G
PRMT	Protein arginine methyltransferase
PRR	Pattern recognition receptor
PTMs	Post-translational modifications
RCD	Regulated cell death
RDA	RIPK1 kinase activity-dependent apoptosis
cFLIPL	Long isoform of cellular FADD-like interleukin (IL)-1β-converting enzyme (FLICE)-inhibitory protein
RHIMs	RIP homotypic interaction motifs
RIPK1	Receptor-interacting protein kinase 1
RIPK3	Receptor-interacting protein kinase 3
RNF146	RING finger protein 146
ROS	Reactive oxygen species
RSK	Ribosomal protein S6 kinase A1
SCF	Skp1-Cullin-1-F-box
SCFSkp2	SCF Cullin-Ring E3 ubiquitin ligase complex
SENP1	SUMO-specific protease 1
SEVO	Sevoflurane
SHARPIN	SHANK-associated RH domain-interacting protein
SIRTs	NAD^+^-dependent sirtuins
Skp2	S-phase kinase-associated protein 2
SMYD2	Protein 2 containing SET and MYND structural domains
SPATA2	Spermatogenesis associated 2
T3SS	Type III secretion system
TAB2/3	TAK1 Binding Protein 2/3
TAK1	TGF activating kinase 1
TAM	Tyro3, Axl, and Mer
TAX1BP1	Tax1-binding protein 1
TBK1	TANK binding kinase 1
TMG	Thiamet-G
TNF	Tumor necrosis factor
TNFRSF	TNF receptor superfamily
TRADD	TNFRSF1A associated via death domain
TRAF2	TNF receptor associated factor 2
TRIF	Toll/IL-1 receptor domain-containing adaptor inducing interferon-beta
Trx1	Thioredoxin-1
*S. typhimurium*	Salmonella typhimurium
Ub	Ubiquitin
UBA	Ubiquitin-associated
UPS	Ubiquitin proteasome system
USP22	Ubiquitin-specific peptidase 22
ZBP1	Z-DNA binding protein 1
ZFP91	Zinc finger protein 91
